# Trophoblast Function in Preeclampsia: Decoding the Mechanistic Roles of Coding and Non-Coding Genes

**DOI:** 10.3390/ijms262311709

**Published:** 2025-12-03

**Authors:** Mihaela Oancea, Stefan Strilciuc, Oana Zanoaga, Cristina Ciocan, Andrei Malutan, Ingrid Păunescu, Dan Boitor, Cornelia Braicu, Dan Mihu

**Affiliations:** 1 2nd Department of Obstetric and Ginecology, “Iuliu Hatieganu” University of Medicine and Pharmacy, 400610 Cluj-Napoca, Romania; mihaelaoancea321@yahoo.com (M.O.); malutan.andrei@gmail.com (A.M.); dan.mihu@yahoo.com (D.M.); 2 Department of Genomics, MEDFUTURE Institute for Biomedical Research, “Iuliu Hatieganu” University of Medicine and Pharmacy, 400347 Cluj-Napoca, Romania; strilciuc.stefan@umfcluj.ro (S.S.); zanoaga.oana@gmail.com (O.Z.); crisciocan@gmail.com (C.C.); 3 1 st Department of Obstetrics and Gynecology, “Iuliu Hatieganu” University of Medicine and Pharmacy, 400347 Cluj-Napoca, Romania

**Keywords:** preeclampsia, trophoblast dysfunction coding, non-coding biomarkers

## Abstract

Preeclampsia (PE) is an obstetric disorder with significant risks to both maternal and fetal health, characterized by hypertension and multi-organ dysfunction. Central to its pathogenesis is the impaired differentiation and function of trophoblast cells, leading to abnormal placental development and defective uterine vascular remodeling. This dysfunctional placentation triggers a cascade of oxidative stress, systemic inflammation, and immune dysregulation, collectively exacerbating disease severity. The trophoblast regulates maternal–fetal interactions through complex and tightly controlled gene expression networks, in which non-coding RNAs such as microRNAs (miRNAs) and circular RNAs (circRNAs) play essential regulatory roles. Here, we summarize current findings on transcriptomic alterations associated with trophoblast anomalies in PE and discuss their potential translational applications. Understanding these molecular mechanisms may enhance early diagnosis, improve clinical outcomes, and pave the way for precision medicine and individualized therapeutic strategies in PE.

## 1. Introduction

Preeclampsia (PE) is a pregnancy-specific disease that usually occurs after 20 weeks of gestation, characterized by the association of hypertension and proteinuria (≥300 mg/24 h or protein/creatinine ratio ≥ 0.3) or hypertension and thrombocytopenia, renal failure, liver disease, neurological symptoms, and pulmonary edema. Poor placentation, resulting from endothelial dysfunction and inadequate trophoblast invasion, is considered a major factor contributing to the development of PE.

PE is characterized by systemic inflammation, which is heightened throughout the maternal circulation and contributes to endothelial dysfunction and multi-organ involvement [1,2,3]. There is considerable interest in identifying biomarkers capable of predicting the onset or progression of PE from the first to the third trimester. However, the limited understanding of the molecular mechanisms underlying the disease and the different evolving forms of PE has significantly limited the identification of reliable biomarkers for early diagnosis and prognosis of the disease [4,5].

Trophoblasts are the embryo’s first differentiated cells, originating from the blastocyst’s trophectoderm. They are responsible for implantation, immune modulation, and vascular remodeling [6]. Trophoblasts initiate implantation by adhering to the uterine lining, and a population of villous cytotrophoblast stem cells is maintained throughout pregnancy. These cells differentiate into two major lineages: the villous and the extravillous (invasive) trophoblasts. The villous lineage, composed of cytotrophoblasts and syncytiotrophoblasts within the chorionic villi, is responsible for placental nutrient and gas exchange, as well as hormone and growth factor production, and contains the developing fetal placental arteries and veins. The extravillous trophoblast lineage includes interstitial and endovascular trophoblasts, which anchor the placenta to the uterine wall and remodel the maternal spiral arteries to increase blood flow to the growing fetus [7]. This process, together with the overexpression of anti-angiogenic factors, leads to widespread endothelial dysfunction and systemic inflammation, with abnormal trophoblast biology playing a major role in the pathogenesis of PE [8].

The failure of extravillous trophoblasts to fully invade and replace the muscular walls of spiral arteries results in high-resistance and low-capacitance vessels, resulting in placental ischemia and fetal hypoxia [8]. These processes are closely associated with the regulation of angiogenic factors, which in turn modulate endothelial function and vascular development [8,9]. Thus, in the first stage, abnormal placental development resulting from defective trophoblast differentiation impairs endovascular trophoblast (EVT) invasion into the uterus, leading to pregnancy complications like preeclampsia. Moreover, insufficient remodeling of spiral arteries leads to placental malperfusion [10,11]. This, in turn, causes progressive oxidative stress in the syncytiotrophoblast (STB), the multinucleated outer layer of the placental villi that serves as the primary site for maternal-fetal exchange and hormone secretion [11]. STB dysfunction is characterized by erosion and damage to its surface, increased shedding of inflammatory factors into the maternal circulation, decreased cell–cell fusion events, and a reduced number of nuclei. Additionally, oxidative stress and inflammation contribute to mitochondrial dysfunction within the STB, further exacerbating cellular injury and disrupting normal placental function [11,12].

Several studies highlight the role of genetic, transcriptomic and epigenetic factors, in modulating trophoblast function and PE [2,13], as can be observed from Table 1. Although numerous models exist to study trophoblast function, none fully replicate them in vivo environment, limiting comprehensive insights into placental development. This shortcoming likely contributes to the slow progress in unraveling the complex mechanisms underlying placental dysfunctions associated with diseases such as PE [14]. Hence, the aim of this review is to summarize key avenues of PE research and highlight the role of trophoblast dysfunction in this complex condition.

**Oxidative stress triggers trophoblast dysfunction in PE.** In early placental development, low levels of reactive oxygen species (ROS) function as key signaling molecules that regulate trophoblast behavior. Controlled ROS production supports trophoblast differentiation by promoting the transition of cytotrophoblasts into syncytiotrophoblasts through enhanced mitochondrial activity [15]. Moderate ROS levels also stimulate the invasive capacity of EVT cells, partly via activation of matrix metalloproteinases (MMPs). Moreover, ROS-mediated signaling contributes to the expression of angiogenic factors such as vascular endothelial growth factor (VEGF), facilitating placental vascular development. In addition, ROS participates in oxygen-sensing pathways that enable the placenta to adapt to fluctuations in oxygen tension during early gestation [15]. However, in PE, excessive ROS generation overcomes antioxidant defenses, leading to oxidative stress that disrupts trophoblast proliferation, invasion, and differentiation [15]. Major sources of ROS in trophoblasts include xanthine oxidase, uncoupled endothelial nitric oxide synthase (eNOS), NADPH oxidase, and mitochondrial dysfunction [15,16].

Mitochondria-derived ROS impair trophoblast fusion and reduce the production of key endocrine hormones, such as human placental lactogen and IGF2, by syncytiotrophoblasts, thereby disrupting placental development and fetal growth. Excess mitochondrial ROS is associated with decreased expression of fusion proteins MFN2 and OPA1 and increased levels of the fission protein DRP1, indicating enhanced mitochondrial fragmentation. These alterations in mitochondrial dynamics compromise syncytiotrophoblast formation and function, while antioxidant treatment with N-acetylcysteine can restore mitochondrial integrity, hormone production, and trophoblast fusion, highlighting the critical role of mitochondrial redox balance in maintaining a healthy maternal–fetal interface [17].

An abnormal ultrastructure and increased ROS levels were correlated with altered unfolded protein response (UPR) pathways in PE compared to healthy control placental tissues [1]. Moreover, invasion and differentiation markers, including syncytin and matrix metalloproteinases (MMPs), were identified with altered expression levels in PE [1]. The role of syncytin-1 in the pathogenesis of PE has been investigated. In this study, it was found that a decrease in syncytin-1 expression may impair the EMT in trophoblast cells by inducing apoptosis and reducing their proliferation and invasive capacity, via inhibition of the PI3K/Akt signaling pathway [18].

These effects were reversed by the antioxidant N-acetylcysteine (NAC), indicating that ROS specifically contribute to trophoblast dysfunction [1]. ROS activates the UPR pathway, particularly through the IRE1α-XBP1 axis [1], a critical mechanism for proper placental development and function [1]. These findings highlight the detrimental impact of ROS-induced UPR on trophoblast cell function, leading to disrupted differentiation and invasion programs. In clinical contexts, these insights suggest that antioxidant therapies may help mitigate trophoblast dysfunction and reduce the onset of PE, offering potential therapeutic options to improve pregnancy outcomes [1]. Maternal immune evasion can be affected by loss of Syncytin1 and Syncytin2 due to ROS, which decreases trophoblast cell fusion [1].

Cellular redox balance is maintained in part using peroxiredoxins (PRDXs), a family of cysteine-dependent peroxidases that detoxify H_2_O_2_, lipid peroxides, and peroxynitrite [19]. PRDX1 was found to regulate trophoblast function via the PTEN/AKT signaling pathway, affecting autophagy and ROS levels, highlighting its potential as a therapeutic target for PE [19]. Mitochondrial ROS disrupt trophoblast function by impairing cell fusion and hormone production, associated with decreased expression of fusion proteins MFN2 and OPA1 and increased levels of the fission protein DRP1, indicating enhanced mitochondrial fragmentation [17]. Scavenging ROS with antioxidants such as N-acetyl cysteine restores mitochondrial dynamics and trophoblast function, highlighting the critical role of redox balance in maintaining a healthy maternal–fetal interface [20].

**Trophoblast and inflammation in PE.** Poor placental perfusion caused by shallow trophoblast invasion results in oxidative stress, which in turn activates inflammation and contributes to endothelial dysfunction [1,21]. In patients with PE, the observed inflammatory milieu is largely driven by the activation of the nuclear factor kappa B (NFκB) signaling pathway [22]. Activation of NFκB alters the cytokine balance toward a pro-inflammatory profile and induces the expression of transcription factors, chemokines, and adhesion molecules, thereby exacerbating the systemic inflammatory state observed in PE [22,23].

In PE, reduced blood flow to the placenta, followed by sudden reperfusion, causes tissue damage and activates inflammation [16]. This process leads to the release of cytokines and inflammatory markers such as TNF-α, IL-6, IL-10, and CRP [16,24]. These molecules can promote oxidative damage and even trigger cell death. A meta-analysis identified a panel of vascular inflammatory gene polymorphisms, TNFα, IL-10, and LPL, as predictors of PE; an increased risk for PE was associated with the VEGF-2578C>A, VEGF-1154G>A, TNF-α-308G>A, IL-10-819C>T, and LPL Ser447Ter variants [25].

Dedicator of cytokinesis 1 (DOCK1) is a guanine-nucleotide exchange factor that activates the small GTPases Rac1 and Cdc42, playing a key role in inflammatory responses, cell phagocytosis, ROS production, and the disruption of cellular barriers [26]. Aberrant DOCK1 expression in the placental villi of PE patients disrupts vascular network formation. DOCK1 deficiency in HTR-8 cells impairs angiogenesis, increases anti-angiogenic ENG expression, reduces VEGF levels, enhances apoptosis, and activates NFκB and pro-inflammatory cytokines (IL6 and TNFα). Mice treated with the DOCK1 inhibitor TBOPP developed PE-like symptoms, underscoring DOCK1’s role in placental dysfunction through inflammation and oxidative stress. These findings highlight DOCK1 as a key factor in PE pathogenesis and a potential therapeutic target [26].

Trophoblast and angiogenesis crosstalk in PE. An antiangiogenic shift, combined with systemic inflammation, leads to endothelial dysfunction, which contributes to the maternal clinical manifestations of PE in its second stage, including hypertension, proteinuria, and organ damage [6,26,27]. A key consequence of trophoblast dysfunction is impaired angiogenesis, characterized by an imbalance between pro- and antiangiogenic factors [28,29,30].

Soluble fms-like tyrosine kinase-1 (sFlt1), known also as soluble VEGF receptor-1 (sVEGFR1), is a circulating anti-angiogenic factor that enhances endothelial dysfunction induced by oxidative stress. The release of this factor has been proven to play a pivotal role in endothelial dysfunction in PE [31]. The primary source for sFlt1 is the placenta, with this factor being associated with endothelial dysfunction and elevated levels observed in women with PE [32]. PlGF, which selectively binds to Flt1, is generally associated with proangiogenic activity under normal conditions. Still, under hypoxic or inflammatory states, such as those observed in PE, it may promote aberrant angiogenesis rather than support normal placental vascular development [33,34]. PlGF and sFlt1 circulate in maternal blood and are described as “angiogenic proteins” because they regulate many facets of arterial vascular function, and they have been proposed as diagnostic markers in pregnancy [35]. The sFlt1/PlGF ratio is a reliable marker for PE, a ratio < 38 rules out the disease, 38–84 indicates high risk, and ≥85 predicts PE and related complications [36].

Endoglin (sEng), the extracellular domain of the homodimeric transmembrane glycoprotein endoglin, represents an antiangiogenic factor released in elevated amounts from the hypoxic placenta in PE [37]. The concentration of this marker was correlated with disease severity, and their overexpression can be detected even before the clinical manifestation of symptoms [37]. Additionally, increased levels of sEng inhibit transforming growth factor-beta (TGF-β) signaling, further compromising vascular integrity [30]. TGF-β1 signaling was found to be regulated by THBS4, whose expression was decreased in PE placental tissue and was localized in trophoblast cells. Knockdown of THBS4 inhibited TGF-β1 pathway activity, reducing trophoblast proliferation, migration, and invasion, effects reversed by a TGF-β1 agonist [38].

Key factors such as VEGF and Eng play a significant role in driving the progression of PE [34]. During pregnancy, VEGF promotes the activation of endothelial nitric oxide synthase (eNOS), resulting in increased NO production [34]. This process mainly signals through the VEGF2-mediated PI3K-AKT signaling pathway, contributing to vascular changes associated with PE [34]. The VEGFA/VEGFR2 signaling pathway was functionally enriched with intronic polyadenylation events, suggesting that post-transcriptional alterations in this angiogenic axis may contribute to the disease’s pathogenesis [39].

Excessive production of anti-angiogenic factors by the dysfunctional syncytiotrophoblast reduces endothelial cell proliferation, migration, and nitric oxide availability, resulting in placental hypoperfusion [40]. Consequently, the disrupted trophoblast–angiogenesis crosstalk contributes not only to placental insufficiency but also to the systemic vascular pathology characteristic of PE [40].

Increased levels of VPO1 and miR-200c were found to play key roles in promoting oxidative stress and inflammatory signaling pathways in PE [41]. Mutually, downregulation of protective peptides, such as humanin and MOTS-c, influences cellular resilience against metabolic and oxidative insults. These changes collectively disrupt the regulation of Eng expression, a key glycoprotein responsible for endothelial cell signaling and vascular integrity [41].

Insulin-like growth factor 1 (IGF1) and its receptor (IGF1R) are crucial regulators of angiogenesis. Dysregulation of the IGF1/IGF1R signaling axis may contribute to abnormal trophoblast invasion and insufficient uteroplacental perfusion observed in PE [42]. Serum and placental levels of IGF1 and IGF1R were downregulated in PE patients versus healthy control samples, and both markers positively correlated with neonatal birth weight [42]. IGF2BP1 enhances the stability of *Neprilysin*, leading to increased expression of this gene and activation of downstream signaling pathways that support trophoblast proliferation, invasion, and angiogenesis. Through this post-transcriptional regulatory mechanism, IGF2BP1 contributes to proper placental development and vascular remodeling, highlighting its potential role in maintaining healthy pregnancy outcomes [43].

Glucose uptake is essential for cellular metabolism, growth, and proliferation, representing the primary substrate for fetal oxidative metabolism. Efficient glucose transport in the placenta is critical for proper fetal development. Among the glucose transporter family, GLUT1 is the most studied; this gene enables glucose transport within the placenta [44]. A recent study revealed that GLUT1 expression is downregulated in placental tissues from women with PE compared with healthy controls. In in vitro studies (BeWo cells), GLUT1 enhances syncytialization by increasing glucose uptake in BeWo cells, stimulating proliferation, migration, and invasion, through MAPK and PI3K/AKT signaling pathways [45].

**Table 1:** Altered coding genes in PE correlated with trophoblast function.

Role	Gene	Expression Level	Observation	Reference
Trophoblast function	GLUT1	↓	Regulated proliferation, migration, and invasion via MAPK and PI3K/AKT signaling	[45]
	Syncytin 1	↓	Attenuates the EMT process by promoting apoptosis, inhibiting proliferation, and invasion by suppressed the PI3K/Akt pathway	[18]
	Follistatin	↓	Key role of FST/GDF11/Smad2/3 signaling axis in trophoblast function regulation	[46]
	THBS4	↓	Key role of TGF-β1/Smad signaling cascade in trophoblast function and placental vascular development	[38]
	SH3PXD2A-AS1	↑	Regulate the invasion and migration of trophoblast cells	[47]
ROS	DOCK1	↓	DOCK1 regulates tube formation and trophoblast angiogenesis; its deficiency promotes apoptosis and ROS production	[26]
	PRDX1	↓	Regulate autophagy and ROS	[19]
	IREα-XBP1s axis		ROS regulated invasion and migration	[1]
Endothelial dysfunction and angiogenesis	ICAM1 and VCAM1	↑	Diagnostic biomarker	[29]
	sFlt-1	↑	Diagnostic biomarker	[32]
	PlGF and sFlt1		sFlt1/PlGF ratio: diagnostic biomarker	[35]
	Htra4	↑	Vascular endothelial injury	[48]
	sEng	↑	Diagnostic biomarker	[37]
	IGFBP2	↑	Regulate trophoblast function and EMT in PE via PI3K/AKT; therapeutic target	[49]
	IGF2BP1 and neprilysin	↑	Potential therapeutic target	[43]
	IGF1 and IGF1R	↓	Biomarkers for the diagnosis and prognosis	[42]
Inflammation	INHBA	↑	Biomarkers for diagnostic and treatment	[50]
	TPBG, and OPRK1	↓		
	NLRP3	↑	miR-223-3p downregulation	[51]
	MAPK1	↑	Differentiating marker between early-onset PE and gestational hypertension	[52]

↑ upregulation; ↓ downregulation.

## 2. MicroRNAs (miRNAs) as Key Regulators of Trophoblast Function and PE Pathogenesis

miRNAs are small, non-coding RNA transcripts (21–25 nucleotides in length) with a role in the regulation of gene expression at the post-transcriptional level [53,54]. A single miRNA can influence multiple genes, while a gene may be regulated by several miRNAs [55]. Therefore, miRNAs were demonstrated to participate actively in implantation and placental development during early pregnancy, playing a crucial role in the pathogenesis of PE [2,56]. Alteration of the expression levels of these transcripts affects key aspects of placentation, including trophoblast proliferation and invasion [56,57], as can be observed from Table 2.

Overexpression of miR-17, miR-20a, and miR-20b has been associated with PE and has been demonstrated to have an important role in placental development by targeting key angiogenic mediators such as ephrin-B2 and EPHB4. Thus, ephrin-B2 and its receptor, EPHB4, are crucial regulators of vascular remodeling and angiogenesis, processes essential for establishing an adequate maternal–fetal blood supply [58]. Dysregulated expression of these miRNAs in preeclamptic placentas disrupts the balance of angiogenic signaling, leading to impaired trophoblast invasion and abnormal vascular remodeling [58].

An in vitro study found that miR-141 is overexpressed in trophoblast cells under hypoxic conditions, being linked to apoptosis stimulation and inhibition of invasion and vascularization. Furthermore, CXCL12β was identified as a direct target of miR-141, suppressing its translation and downregulating MMP2, p62, and LC3B, while promoting upregulation of ROCK1 and RhoA. Arachidonic acid reverses CXCL12β-mediated effects, restoring invasion and reducing apoptosis. Hypoxia-induced miR-141 disrupts trophoblast function by suppressing the CXCL12β and CXCR2/4 pathways and impairing placental development [59].

MiR-3935 has a crucial role in promoting EMT in trophoblast cells and was found to be downregulated in placental and peripheral blood samples from patients with PE. Trophoblast EMT is negatively regulated through the inhibition of RGS2, controlling the methylation status of the CDH1 gene promoter; this mechanism involves the stimulation of ALKBH1 and overexpression of E-cadherin, a protein encoded by the CDH1 gene. The elevated levels of E-Cadherin hinder proper trophoblast EMT, contributing to the pathological development of PE [60].

Decidua-derived mesenchymal stem cells (DMSCs) were used to study the expression level of miR-16; in this study, miR-16 was found to be overexpressed and was linked to cell proliferation and migration, and stimulation of cell-cycle arrest by targeting cyclin E1 [61]. Furthermore, miR-16 upregulation reduced the cells’ capacity to induce blood vessel formation by human umbilical vein endothelial cells (HUVECs). Also, an inverse correlation between miR-16 expression and VEGF-A and cyclin E1 levels was identified, suggesting that altered miR-16 expression in DMSCs may contribute to the pathogenesis of PE [61].

Decorin (DCN) is a proteoglycan produced by chorionic villus mesenchymal and decidual cells, which targets tyrosine kinase receptors and inhibits trophoblast functions such as proliferation, migration, invasion, and endovascular differentiation [62]. Recent research identified that miR-512-3p inhibits extravillous trophoblast functions by modulating the USF2/PPP3R1 axis. Specifically, miR-512-3p impairs trophoblast migration, invasion, and VEGF-dependent differentiation, while paradoxically increasing PPP3R1 expression, a known target, through suppression of USF2, a transcription factor regulating PPP3R1 [63].

Knockdown of miR-31-5p was found to partially counteract the promotive effects of SNHG5 silencing on autophagy in trophoblast cells, indicating a complex regulatory relationship between these transcripts. Further analysis revealed that miR-31-5p is a downstream effector in this regulatory axis. These findings provide valuable insights into the molecular mechanisms governing trophoblast autophagy, highlighting the interplay between lncRNAs and miRNAs in placental biology [64].

In vitro and in vivo studies revealed that let-7a was highly expressed in early-onset severe PE [65]. Thus, in JEG-3 cells, let-7a overexpression was associated with a decrease in cell viability, promotion of apoptosis, and cell cycle progression. Bcl-xl and YAP1 are two target genes that have been identified in this study with the ability to protect against the apoptosis induced by let-7a. Furthermore, demethylation of let-7a was associated with their increased level. This study also revealed a decrease in tumorigenesis in vivo animal models. These findings demonstrated that let-7a induces trophoblast apoptosis via a mechanism involving the downregulation of Bcl-xl and YAP1 [65].

The potential of serum miR-515-5p as a biomarker for PE was demonstrated in a study using HTR-8/SVneo trophoblast cells. This study revealed that inhibition of cell proliferation and invasion was linked to upregulation of miR-515-5p via inhibition of XIAP [66]. An interesting in vitro study revealed that migration, invasion, and tube formation of trophoblast cells are inhibited by miR-210, which was found to be overexpressed, leading to the activation of apoptotic signaling, through downregulation of NOTCH1 [67].

The migration and invasion of trophoblast cells were stimulated by downregulation of miR-95-5p, while miR-95-5p upregulation was linked to the expression level of MMP9, TIMP1, and MMP2 by targeting low-density lipoprotein receptor-related protein 6, encoded by the LRP6 gene [68].

An inverse correlation between miR-149-5p and endoglin (ENG) expression was found in the placenta from PE patients, with ENG being a direct target of miR-149-5p. miR-149-5p increases the invasion of trophoblast cells, and upregulation of sEng abrogates this stimulation [69]. Recent studies demonstrated the contribution of miR-18a to the development of PE, with its overexpression stimulating trophoblast cell invasion and suppressing Smad2 activation by transforming growth factor-β (TGF-β) [70]. Upregulation of miR-146a-5p was identified in the placentae from PE patients. Moreover, in vitro experiments confirmed that miR-146a-5p inhibited trophoblast cell progression, invasion, and migration through inhibition of Wnt2 [71].

**Table 2:** Altered miRNAs in PE related to trophoblast function.

miRNA	Expression Level in PE	Observation	Reference
miR-16	↑	Regulate proliferation and angiogenesis of mesenchymal stem cells in promoting PE; negatively regulated by Cyclin E1 and VEGFA	[61]
miR-31	↑	Regulate trophoblast autophagy via SNHG5	[64]
miR-17, miR-20a, miR-20b	↑	Regulate trophoblast and endothelial cells angiogenesis through EPHB4 and ephrin-B2	[58]
miR-141	↑	Regulate apoptosis, invasion, and vascularization by inhibiting CXCL12β/CXCR2/4 in trophoblast	[59]
miR-296	↑	Regulate endothelial dysfunction and trophoblast invasion	[72]
miR-454	↓	Promote trophoblast cell proliferation, apoptosis, and invasion via EPHB4	[73]
miR-512	↑	Regulate migration and invasion of trophoblast cells via USF2/PPP3R1 axis	[63]
miR-3935	↓	Regulate trophoblast EMT through miR-3935/TRAF6/RGS2 axis	[60]
Let-7a	↑	Stimulate trophoblast apoptosis via Bcl-xl and YAP1	[65]
miR-515-5p	↑	Inhibit trophoblast cell proliferation and invasion	[66]
miR-210	↑	Stimulate apoptosis of trophoblast via NOTCH1	[67]
miR-95-5p	↑	Regulate migration and invasion of trophoblast cells	[68]
miR-149-5p	↑	Inhibit trophoblast Eng expression	[69]
miR-18a	↑	Regulate inhibition of trophoblast cell invasion by transforming TGFβ	[70]
miR-146a-5p	↑	Inhibit trophoblast cell progression, invasion, and EMT	[71]

↑ upregulation; ↓ downregulation.

## 3. Circular RNAs Linking Abnormal Trophoblast Function to PE

Circular RNAs (circRNAs) are a class of non-coding RNAs characterized by their covalently closed loop structures, which confer increased stability compared to linear RNAs [74]. Emerging research has highlighted their significant roles in the trophoblast function [75,76], as can be observed from Table 3.

A critical regulator in the pathogenesis of early-onset PE is circRNA_06354. It was found to suppress trophoblast invasion, migration, and tube formation toward the endometrium, leading to impaired placental development. Furthermore, circRNA_06354 have a significantly higher expression level compared to its linear isoform. Emerging evidence suggests that the circRNA_06354/miR-92a-3p/VEGF-A axis modulates angiogenesis and vascular remodeling during early pregnancy. This represents a promising therapeutic target, with potential applications in preventing and treating early-onset PE through by restoring normal trophoblastic function and enhancing placental vascularization [77].

Notably circ_0001326 was identified as a regulatory factor for HTRA1 through its ability to sequester miR-188-3p, thereby modulating its activity. This interaction highlights a potential regulatory axis involving circRNA, miRNA, and mRNA in trophoblast cell function. To further investigate this mechanism, a series of rescue experiments was conducted, which revealed that miR-188-3p could reverse the inhibitory effects caused by circ_0001326 knockdown on trophoblast cells, including proliferation, migration, and invasion. Furthermore, inhibition of HTRA1 significantly reduces the effects of the miR-188-3p inhibitor on trophoblast cell phenotype alterations, proving the interplay between circ_0001326, miR-188-3p, and HTRA1 in the regulation of trophoblast cell behavior [78].

Additionally, circ_0090100 promotes the expression of AHNAK by sponging miR-139-5p. This interaction inhibits trophoblast cell proliferation and invasion while promoting apoptosis, contributing to the placental dysfunction characteristic of the PE [79]. Another apoptotic regulator, circ_0017068, functions by sponging miR-330-5p and modulating the expression of the XIAP gene [80].

Notably, circ_0008726 has potential as a biomarker for PE. It was found to be upregulated in PE and influenced placental development by inhibiting trophoblast invasion, migration, and EMT through modulation of the miR-345-3p/RYBP axis [81]. Similarly, circ_0015382 regulates the same mechanisms but via the miR-149-5p/TFPI2 axis, enhancing the role of these pathways in the pathogenesis of PE [82].

Overexpression of circ_0014736 was revealed in PE placenta tissues, alongside GPR4, while miR-942-5p was downregulated in PE samples compared to normal placentas. Functional validation demonstrated that downregulation of circ_0014736 stimulates proliferation, invasion, and migration of trophoblast cells (HTR-8/SVneo) and suppresses apoptosis, highlighting its role as a negative regulator of trophoblast function in PE via the circ_0014736/miR-942-5p/GPR4 axis [83].

In addition, circ_0063517 and ETBR (Endothelin B Receptor) were found to be significantly downregulated, while miR-31-5p was upregulated in PE placental tissue. Overexpression of circ_0063517 or downregulation of miR-31-5p promotes vascular endothelial cell proliferation, migration, and angiogenesis. Furthermore, circ_0063517 regulates ETBR and VEGFA/VEGFR2 expression. The role of circ_0063517 in promoting angiogenesis was confirmed in vivo, supporting the key role of the circ_0063517/miR-31-5p/ETBR axis in the regulation of placental angiogenesis in PE [84].

Circ_0077109 was significantly upregulated in PE and contributes to impaired placental function. Its overexpression suppresses trophoblast proliferation, invasion, and angiogenesis while promoting apoptosis. Mechanistically, circ_0077109 acts as a sponge for miR-139-5p, thereby reducing its availability. MiR-139-5p normally targets HOXD10, a gene whose overexpression similarly inhibits trophoblast function. Furthermore, miR-139-5p inhibition reduces trophoblast activity, an effect that can be reversed by HOXD10 knockdown, confirming the involvement of the circ_0077109/miR-139-5p/HOXD10 axis in the pathogenesis of PE [85].

**Table 3:** Altered circRNAs in PE related to trophoblast function.

miRNA	Expression Level	Target Biological Process	References
circ_0090100	↑	Regulate trophoblast cell proliferation, invasion, and apoptosis through circ_0090100/miR-139-5p/AHNAK axis	[79]
circ_0017068	↓	Regulate trophoblast proliferation and apoptosis via miR-330-5p/XIAP axis	[80]
circ_0001326	↑	Stimulate trophoblast cell proliferation, invasion, migration, and EMT via miR-188-3p/HTRA1 axis,	[78]
circ_0008726	↑	Promote trophoblast migration, invasion, and EMT by miR-345-3p/RYBP signaling axis	[81]
CircRNA_06354	N/A	Regulate angiogenesis	[77]
circ_0014736	↑	Inhibit trophoblast proliferation, migration and invasion via miR-942-5p/GPR4 axis	[83]
Circ_0015382	↑	Inhibit cell growth, invasion and migration via miR-149-5p/TFPI2 axis	[82]
Circ_0063517	↓	Regulate angiogenesis	[84]
circ_0077109	↑	Inhibit cell proliferation, invasion, and angiogenesis via miR-139-5p/HOXD10 axis	[85]
circ_0007445	↑	Suppress trophoblast cell function via miR-4432/HTRA1 axis	[86]
hsa_circ_0002348	↑	Inhibit cell proliferation and promote apoptosis	[87]
circPAPPA2	↑	Inhibit cell proliferation and invasion	[88]
circ_0030042	↑	Regulate cell growth, invasion, and EMT process	[89]
circUBAP2	↓	Inhibit cell proliferation and migration	[90]

↑ upregulation; ↓ downregulation, N/A, not available.

## 4. Conclusions

The disrupted transcriptomic profile observed in PE reflects alterations in trophoblast function, which plays a central role in the pathogenesis of the disease. Dysregulation of key genes, miRNAs, and circRNAs impacts trophoblast differentiation, invasion, and the remodeling of spiral arteries, leading to inadequate placentation and subsequent maternal endothelial dysfunction. These transcriptomic alterations mirror the molecular imbalances driving inflammation, oxidative stress, and immune dysregulation in PE. Understanding these changes not only develops our knowledge of trophoblast-related mechanisms but also opens new avenues for the identification of biomarkers and targeted therapeutic strategies aimed at improving maternal and fetal outcomes.

## Data Availability

No new data were created or analyzed in this study.
